# Immunotherapy as a Turning Point in the Treatment of Melanoma Brain Metastases

**DOI:** 10.15190/d.2023.8

**Published:** 2023-06-30

**Authors:** Gil Nuno Castro Fernandes

**Affiliations:** ^1^Department of Neurological Surgery, Sion Hospital, Switzerland

**Keywords:** Melanoma, brain metastasis, immunotherapy, clinical trial.

## Abstract

The incidence of tumor metastases in the brain is many times more frequent than primary brain tumors, affecting a very large share of patients suffering from systemic cancer. Advanced malignant melanoma is well known for its ability to invade the brain space and current treatment options, such as surgery and radiation therapy, are not very efficient and cause notable complications and morbidity. The aim of this review is to explore the recent advances and future potential of using immunotherapy in the treatment of melanoma brain metastases. Several FDA approved immunotherapeutic drugs have shown to be able to at least double the overall survival rates in such patients. Clinical trials of varying phases are underway and available results are promising, significantly prolonging survival rates in patients with previously untreated melanoma brain metastases. Nevertheless, not all patients respond to these immunotherapies, facing a high percentage of resistant cases, without yet knowing the mechanisms and causes of resistance behind. Also, at the time of immunotherapy, a small percentage of patients is affected by pseudoprogression, which can be difficult to distinguish from true progression given the similarity of symptoms. Therefore, there is a pressing need for future research about treatment effectiveness in patients with brain metastases from melanoma, including outcomes from the perspective of patients.

## 1. Introduction

Brain metastasis, the spread of a tumor from a primary neoplasm to the brain, is about 10 times more frequent than a primary brain tumor^1^. Most common brain metastases have their primary tumor in the lung (~45%), breast (20%) and skin (e.g., melanoma, 10%)^2^. Brain metastases have a very poor prognosis and are characterized by a progressive central nervous system (CNS) damage and functional decline, significantly affecting quality of life and shortening survival rates. Advanced melanoma is well known for its potential to metastasize to the brain. However, current therapies are not very efficient and brain metastases are in most cases lethal.

Treatment of melanoma brain metastases with surgery and/or radiation therapy results in median overall survival of only about 4 to 6 months after diagnosis^3^ and they cause notable complications and morbidity (stroke, radiation-induced necrosis and cognitive defects)^4^. New immunotherapies, such as the targeted or immunomodulatory drugs, many in clinical trials, have shown promise, with some immunomodulatory drugs being able to at least double the overall survival rates in melanoma brain metastases patients^5^. Immunotherapy uses components of the body’s own immune system to fight against cancer. It works in several ways, for example by enhancing the capacity of the immune system to attack cancer cells or giving the immune system specific components artificially produced^6^. In particular, immunomodulators, antibodies stimulating T-cell function either by blocking or activating regulatory receptors, have been shown to cause regression of several types of tumors and an exponential number of clinical trials is underway. Remarkably, several immunomodulatory drugs/checkpoint inhibitors are already approved by the US Food and Drug Administration (FDA) for the treatment of melanoma, non-small cell lung cancer, breast cancer, bladder cancer, kidney cancer, and Hodgkin lymphoma^7,8^.

## 2. Epidemiology of Malignant Melanoma

Malignant melanoma is the most life-threatening and deadly type of skin cancer, representing approximately 5-10% of all skin-cancers, but being responsible for more than 80% of deaths related to skin-cancer^9-11^. The other representants of skin cancer are basal cell carcinoma (BCC), squamous cell carcinoma (SCC) and Merkel cell carcinomas^9^.

Recent data have shown that worldwide incidence of melanoma has been rising, making it the fifth most common type of cancer in adults, the first, second, third and fourth places being respectively occupied by breast cancer, lung and bronchus cancer, prostate cancer, and colon and rectum cancer^10,12,13^.

Risk factors linked to melanoma development have been identified, including intense exposure (acute-intermittent rather than chronic) to sources of ultraviolet radiation (either natural – sunlight; or artificial – tanning bed), genetic predisposition, positive family history, compromised immune system, obesity, exposure to heavy metals and some pesticides, and alcohol consumption^9,13,14^.

Outstandingly, amid all solid tumors, melanoma has the highest tendency for brain metastases^15,16^.

## 3. Malignant Transformation of Melanocytes

Melanoma’s cellular origin has been an important focus of research because of its doubtfulness. However, a recent study led by Kohler et al. has demonstrated that melanoma can arise from pigment-producing melanocytes residing in the interfollicular layer of epidermis^17^.

One of the valuable roles of melanin is the creation of a sunshield protecting basal melanocytes from DNA damage induced by ultraviolet radiation^18^. Nonetheless, if DNA impairment occurs and remains unrepaired, it can trigger mutations in the pigment-producing melanocytes, leading them to quickly multiply and undergo malignant transformation through a chain of reactions known as melanomagenesis^19^. The first stage in this process is the development of nevomelanocytes (an accretion of pigment cells) of benign/common nevi, which are cells characterized by atypical proliferation and arrested progression due to cellular senescence (a steady cessation of cell division occurring in response to several intrinsic and extrinsic factors despite the presence of mitogenic signals and optimal growth conditions)^14,18,20^. The second stage is the overriding of cellular senescence by enhancing both the cell cycle and the length of telomere^9^. This is one of the critical shifts leading to dysplastic nevi, which are cells characterized by atypical qualities and carrying the risk for melanoma development^10,14^. The third stage can be divided into two progressive phases: radial and vertical. The radial phase is characterized by an outward proliferation of melanoma cells, allowing them to spread across the epidermis or invade the papillary dermis. The vertical stage is characterized by the invasion of the dermis and the ability to disseminate or metastasize throughout the body^10,14,19^. The metastatic cells will first invade and proliferate at local or regional sites (e.g., regional lymph nodes) and then at distant sites, the most common being lung, liver, distant areas of the skin, brain, gastrointestinal tract, bone and adrenal gland^19^. The progression between successive stages of melanomagenesis is thought to be driven by the simultaneous accumulation of genetic, epigenetic and allogenetic variations^9-11^. Even though this model has been commonly accepted as a reference for the development of malignant melanoma, recent findings based on epidemiological, clinical and experimental data reveal that it only applies to a third of melanoma cases, thus evidencing that melanoma development might be more complicated and less stepwise as originally thought^10,14^.

Malignant melanoma is the tumor with the highest number of mutations^10,21^. Wide-ranging cytogenetic and high-resolution genomic analysis have shown that genetic variations exponentially increase as it progresses from nevus to primary and later to metastatic melanoma^14^. Thus, a number of key genes and pathways have been revealed to play a role in melanoma development, progression and proliferation, ranging from signal transduction to developmental and transcriptional pathways and cell cycle deregulation. Several mutations, known as driver mutations (BRAF, NRAS, KIT, GNAQ, GNA11, NF1, and TERT), define most of the molecular subtypes of melanoma. However, studies have shown that these mutations alone are not enough to develop a straightforward tumorigenic phenotype. They require the presence of the so-called “supporting mutations”. It is therefore important to keep searching for mutations (both driver and supporting) in melanoma in order to identify new molecular subtypes and, ultimately, guide targeted therapy choices to achieve long-lasting responses^11,22-26^.

Although some key genetic stimuli are needed for melanomagenesis to occur, alone, they are not enough. Years of research have demonstrated that a synergetic interaction between environmental, genetic, and host factors is of vital importance for the malignant transformation of melanocytes. Tumor microenvironment is a complex and dynamic setting in close interaction with several structures, notably extracellular matrix, fibroblasts and microvasculature. It modulates the transformation process by influencing the concentration of key factors necessary for tumor cells to grow, these including growth factors, cytokines, nutrients (e.g., glucose), and metabolic gases (e.g., oxygen). Therefore, tumor microenvironment can either increase or decrease the likelihood for melanomagenesis to occur^11^.

## 4. Melanoma Brain Metastases Origin and Development

### 4.1 Transmigration of Melanoma Cells to the Brain

**Figure 1:**
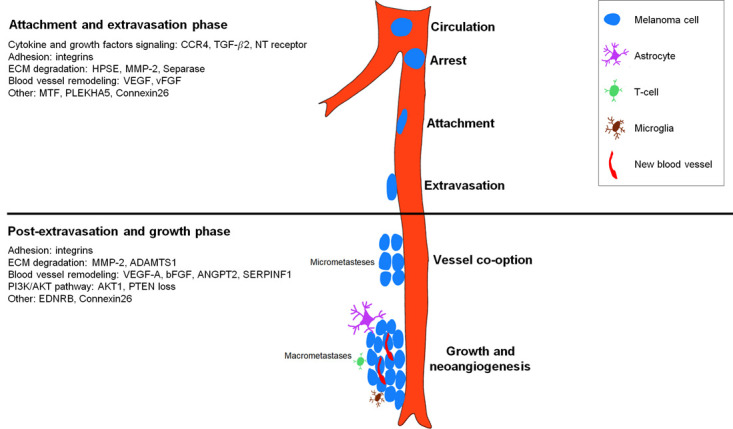
Process of Melanoma Transmigration to the Brain The metastatic cascade to the brain involves a series of steps allowing melanoma cells to disseminate from their primary site to the brain parenchyma: 1) Cell inflowing to the blood circulation at primary site, 2) Cell arrest in the microvasculature, 3) Adhesion to the endothelial cells. 4) Extravasation to the brain parenchyma, 5) Vessel co-option allowing the formation of micromestases. 6) Growth and neoangiogenesis empowering the magnification from micrometastases to macrometastases.

Studies have shown that metastatic melanoma cells have evolved from their primary site and have acquired a selective brain tropism, thus enabling them to establish secondary neoplasms within the brain^27^. The mechanisms through which melanoma cells disseminate to the brain have remained unclear over the years, however the development of *in vivo*, live-cell imaging techniques provided new understandings about the underlying processes involved^16,28^ (Figure 1). In the initial phase of the metastatic cascade to the brain, melanoma cells enter the circulation and then undergo hematogenous spread towards the brain vasculature, where they arrest upon reaching the narrow blood vessel branch points and capillaries of the microvasculature^15,16^. There, they linger in an inert state for about 1–9 days (significantly slower cell migration when compared to that of other organs, which might explain the latency of melanoma brain metastasis development), allowing their adhesion to the endothelial cells^16^. In order to extravasate from the blood-brain barrier into the brain parenchyma, metastatic cells need to 1) push the endothelial cells apart through the action of mechanical forces (they become round cell and develop cytoplasmic protrusions), 2) disrupt tight junctions through the action of pro-invasive integrins (31, b3, 41) and proteases (cathepsin-S), and 3) degrade the basement membrane through the action of proteases (matrix metalloproteinases 2 and 9, heparanases)^15,16^. Studies have shown that some conditions might facilitate the transmigration process of melanoma cells, notably their affinity for soluble solutes produced within the brain (e.g., growth factors, cytokines) and shared transcriptomic lineage with brain cells. In fact, melanoma cells exhibit neurotrophin receptors with a high affinity for neurotrophins produced from the brain, indicating that these substances may play a role in their recruitment^15,27^. Once inside the brain parenchyma, melanoma cells initiate a vessel co-option and remain closely associated with the endothelial cells at the abluminal surface, where they start forming micrometastases and further invade the brain^15,16^. It has been observed that melanoma cells that did not have any contact with the blood vessels quickly die^16,27^. The brain microenvironment (notably astrocytes, microglia and T-cells) then influences the growth from micrometastases to macrometastases^15^. Further growth of metastases might involve the formation of new blood vessels at the tumor margin (neoangiogenesis)^15,16^. Mysteriously, some individual melanoma cells stay in a dormant state while co-opting the brain microvasculature, but still possessing the ability to migrate along it^16^.

### 4.2 Brain Tumor Microenvironment

Tumor microenvironment is an important factor influencing all steps of metastasis development, from metastasis formation to its progression and response to different therapies, by providing pro-tumorigenic signals. These signals could be intrinsic or produced and secreted as a response to the metastatic process itself. Either way, they support viability, growth and proliferation of metastatic cells at secondary sites. In addition to the tumor cells, other types of cells can be found in the brain tumor microenvironment, including fibroblasts, immune cells, pericytes, and endothelial cells^27^. The main features distinguishing the brain tissue from any other tissues are the presence of blood-brain barrier (BBB) and unique resident cells (microglia, astrocytes and neurons), a distinctive immune advantage, and very high nutritional demands and energy consumption^27,29^.

### 4.3 Blood-Brain Barrier 

The blood-brain barrier, unique to the CNS, is located at the level of cerebral capillaries, and is a highly selective multicellular layer, that protects neural cells by restricting free movement of substances and cellular elements between the systemic circulation and brain tissue. Its exceptional structure is composed of tight junctions, which are dynamic arrangements located between endothelial cells and formed by transmembrane (occludins, cadherins, claudins and junctional adhesion molecules) and cytosolic (catenins and zonula occludens) proteins^15,30-32^. Under physiological conditions, this semipermeable membrane only allows the passage of certain substances, either by passive diffusion (e.g., water, lipid-soluble molecules and gases) or active transport (e.g., nutrients, other molecules)^30^. A group of specific cells, namely endothelial cells, pericytes, astrocytes, microglia and neurons, forms the neurovascular unit, which regulates and supports tight junctions in a synchronized and coordinated manner^30,32^. Some studies suggest that the blood-brain barrier is compromised throughout the course of the metastatic proliferation to the brain, thus allowing the passage of certain substances, otherwise not possible under physiological conditions^15,30-42^. Some additional elements, particularly active transporters, adsorbent endocytosis and vesicular pathways, also contribute to the physiological function of the blood-brain barrier, however their role in the metastatic process is poorly recognized, thus evidencing the need for further studies^30^. In the setting of brain metastases formation, the blood-brain barrier holds a binary function: it protects the central nervous system from incoming cancer cells, but it also protects metastatic cells by supporting their transmigration, proliferation and survival inside the brain. In fact, after crossing the blood-brain barrier, metastatic cells escape the immune surveillance, and their growth is further potentiated by elements secreted by the barrier itself^32^.

### 4.4 Interaction with Brain Parenchyma Cells 

**Figure 2:**
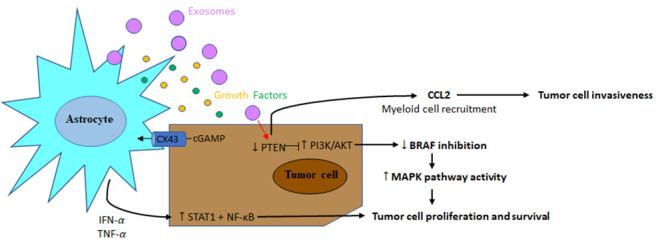
Pathways activated upon interaction between Astrocyte and Tumor cell Second messenger cGAMP activates the STING pathway in astrocytes, thus allowing the release of specific cytokines triggering STAT1 and NF-κB-mediated survival and/or proliferation of tumor cells. The secretion of exosomes and growth factors silences PTEN, leading to the occurrence of two important phenomena: 1) the release of CCL2 from tumor cells, allowing myeloid cell recruitment via CCR2, which in turn promotes the chemotaxis and chemokinesis of tumor cells, and therefore tumor cell invasiveness; 2) activation of the PI3K/AKT pathway in melanoma cells, successively limiting their inhibition by BRAF kinase, which in turn upregulates the MAPK pathway, therefore their proliferation and survival.

Once inside the brain, melanoma cells come into contact with multiple cell types and their interaction can have either tumor-suppressive or tumor-supportive effects^16^. Astrocytes represent roughly 50% of all cells in the brain and have an indispensable role in homeostasis. They support repair of brain tissue following injuries, and support endothelial cells in obstructing melanoma cells from entering the brain. Nevertheless, astrocytes are the most frequent cells implicated in brain metastasis development. They become activated upon interacting with tumor cells and start to secrete many soluble factors that support metastasis proliferation and survival in the brain microenvironment^m^ (Figure 2).

The most well-known soluble factors secreted by the so-called reactive-astrocytes include neurotrophins (growth factors), chemokines and cytokines (IL-6, TNF-α, IL-1, and IL-23). Remarkably, it has been shown that reactive-astrocytes also have the aptitude to induce the expression of several pro-survival genes (e.g., TWIST, BCL2L1) and extracellular matrix (ECM) degrading enzymes (e.g., metalloproteinases 2 and 9, heparanase) in tumor cells. Protocadherins and Connexin-43 (Cx43)-mediated gap junctions, where the transfer of the second messenger cytosolic guanosine–adenosine monophosphate (cGAMP) activates the STING pathway in astrocytes and instructs them to produce and secrete tumor-stimulating cytokines (e.g., INF-α, TNF-α) are thought to be the means by which tumor cells from brain metastasis communicate with local astrocytes. These cytokines will then promote STAT1 and NF-κB-mediated survival and/or proliferation of cancer cells^16,17,27,30^. Gap junctions can be successfully targeted^33^. Astrocytes also regulate tumor cell survival by means of epigenetic changes. They silence PTEN, a major tumor suppressor, by secreting exosomes containing micro-RNA-19a. This silencing will then activate the release of C-C motif chemokine ligand 2 (CCL2) from tumor cells, allowing myeloid cell recruitment via their C-C chemokine receptor type 2 (CCR2), which in turn will promote the chemotaxis and chemokinesis of tumor cells, and therefore tumor cell invasiveness^34^. Mediator PTEN is also a major regulator of the PI3K/AKT signaling pathway, and it has been shown that reduced PTEN expression is accompanied by elevated PI3K/AKT pathway activity in melanoma cells, which additionally limits their inhibition by BRAF kinase, thus upregulating the MAPK pathway (also known as the RAS/RAF/MEK/ERK signaling cascade) and subsequently promoting their proliferation and survival^16,17,27,30,35^. A recent retrospective analysis has also shown that concomitant occurrence of PTEN silencing with BRAF V600E mutation (the most common mutation in metastatic melanoma, which activates MAPK-ERK signaling pathway) revealed an earlier development of brain metastasis and consequently a shorter overall survival^16^. Microglia are the innate immune cells in the brain and resemble peripheral macrophages. They possess both tumor-suppressive and tumor-supportive effects. The main tumor-suppressive effects involve cell cytotoxicity mediated by nitric oxide, tumor cell phagocytosis, and activation of tumor-specific B- and T-lymphocytes, while the main tumor-supportive effects involve expression of programmed death-ligand 1 (PD-L1) leading to inhibition of cytotoxic T-cells, and secretion of factors inducing cancer growth and invasion (e.g., growth factors, chemokines)^15,16,30^. There is ongoing research to determine if these cells are also involved in the Wnt-signaling pathway leading to metastasis invasion and colonization of the brain^15^. T-cells, also called T-lymphocytes, are major components of the adaptive immune system. Some subgroups exhibit tumor-suppressive effects (notably effector CD3+, cytotoxic CD8+ and memory CD445RO+), while some other subgroups exhibit tumor-supportive effects (regulatory FoxP3+ and immune tolerance PD-1+). Although their presence in brain parenchyma is quite rare under physiological conditions, it has been previously establishedthat melanoma brain metastases expressing PD-L1 have a higher infiltration of T-cells^15^. Furthermore, higher density of CD3 and CD8 tumor-associated lymphocytes has been correlated with increased survival^43^. Taking into consideration these results, it makes sense to consider immunotherapy as a potentially promising tumor-targeting strategy in melanoma brain metastases. Recent clinical trials have confirmed this hypothesis to be correct.

### 4.5 Risk Factors and Metastasis Distribution

A set of risk factors has been explicitly linked to the development of melanoma brain metastases, including: male gender, age over 60 years, primary disease from mucosal surfaces or skin of the head, neck, scalp or trunk; acral, lentiginous, or nodular tumor histology; high Clark’s level/Breslow thickness of the primary disease; occurrence of visceral or nodal metastases; unknown primary melanomas; increased serum lactate dehydrogenase (LDH) levels; presence of oncogenic BRAF and NRAS mutations; expression of CCR4 on melanoma cells; and activation of PI3K/AKT signaling pathway^16,31,44^. Melanoma brain metastases are the most frequent intracranial tumors in adults^27^ and their location within the brain is well correlated with those areas receiving the highest blood flow: cerebral hemispheres (80%, from which 43.5% are located within the frontal lobe), cerebellum (15%) and brain stem (5%)^16,31,45^.

## 5. Therapeutic Strategies for Melanoma Brain Metastases

Magnetic Resonance Imaging (MRI) is the gold standard for both diagnosis and monitoring of brain metastases^46^. According to the TNM (Tumor, Node, Metastasis) staging system, patients with melanoma can be clinically divided into three groups: patients with 1) local disease (stage I–II), 2) node-positive disease (stage III), and 3) advanced or metastatic disease (stage IV)^47^. When devising a therapeutic strategy in certain patients with melanoma metastases, important issues about therapeutic repercussions must be considered following prolonged survival and long-term remissions. As a result, for the correct treatment of a patient with brain metastases, a multidisciplinary strategy that examines all possible treatment modalities is required. For the correct designing of a comprehensive therapeutic approach, important aspects need to be considered, notably the clinical features of brain metastases (e.g., number, size, location, and extent of CNS symptoms), extracranial systemic disease, presence of BRAF mutation, patient performance status (patient’s ability to perform daily activities without any help), associated comorbidities, and prior exposure to intracranially effective therapy (e.g., immunotherapy, BRAF/MEK inhibitors, stereotactic radiosurgery)^44,48^. In earlier times, the only options to control brain metastases were locoregional therapies such as surgical excision and/or radiation therapy (whole-brain radiation therapy - WBRT, and stereotactic radiosurgery - SRS)^44,49^. In addition to being generally inefficient, with a median overall survival of only 4-6 months following diagnosis, they also cause notable complications and morbidity^4^. Method WBRT is the standard treatment for metastatic brain tumors, with WBRT and surgical removal being used for multiple and/or large tumors and MRI-assisted SRS for smaller tumors. Tumor Treating Fields method is an additional option used in treating brain metastasis^50,51^. Focal therapies such as SRS and surgery are limited to the treatment of the area of interest, which may result in tumor relapse from other non-treated sites which were under the limit of detection of available imaging methods^52^. In general, SRS is preferred to WBRT in the treatment of melanoma brain metastasis^53^. Melanoma cells usually have a powerful DNA damage repair machinery, thus resulting in the need of delivery of larger fractions/doses of radiotherapy^54^. Chemotherapy was previously the only approved medication for metastatic melanoma, but the results in melanoma patients with brain metastasis were disappointing, similar to those obtained in melanoma treatment in general, with only 5-20% of patients having their tumor shrink, but no improvement in overall survival, despite it^55^. In recent years, the development of new systemic treatment modalities such as immune check point inhibitors, and BRAF plus MEK inhibitors provides an alternative for patients suffering from melanoma brain metastases by virtue of their intracranial efficacy^44^. FDA-approved targeted therapies such as vemurafenib, trametinib, dabrafenib, and some of their combinations, act by blocking BRAF with activatory mutations such as V600E or V600K^56,57^. However, in spite their intracranial efficacy, resistance develops in the majority of treated cases. The occurrence of resistance in melanoma brain metastases is poorly understood, and the specific CNS microenvironment may contribute to different resistance mechanisms than those previously described in extracranial melanoma^58,59^. Remarkably, immunotherapy has demonstrated tremendous promise, being able to at least double the overall survival rates for patients with melanoma brain metastases^5^ Outstandingly, radiation has the ability to enhance these treatments^60^, while also reducing their side effects (e.g., neurotoxicity)^43^.

## 6. Immunotherapy as a Turning Point in the Treatment of Melanoma Brain Metastases

### 6.1 Definition

The regulation of the immune system is a highly complex process. It involves a multitude of components, one of these being immune checkpoints, which are responsible for self-tolerance, the immune system's ability to recognize what is ‘self’ and not react against or attack it^61,62^. Immune checkpoint inhibitors, like anti-PD-1/PD-L1/CTLA-4, are a form of immunotherapy regulating this process by boosting immune reactions against tumor cells, while also endorsing autoimmunity. Through the action of interferon gamma, these molecules are upregulated by the inflammatory response^63-65^.

### 6.2 Mechanism of action

Research studies have demonstrated that CD4 and CD8 lymphocytes are required for limitation or prevention of brain metastasis, with an important role assigned to the regulatory T-cells (Treg)^66^. The most important molecules as immune checkpoints are the programmed cell death protein 1 (PD-1) and its ligand programmed death-ligand 1 (PD-L1) and the cytotoxic T-lymphocyte-associated protein 4 (CTLA-4). Protein PD-1, also known as CD279, is mostly found on the activated CD8+ T-cells, but also on the surface of dendritic cells, macrophages, and B-cells. Despite of its similarity to CD28, it interacts with its specific ligands: 1) PD-L1, which is expressed on the surface of various cells, including hepatocytes, myocytes, cancer cells, immune cells, pancreatic islet cells, endothelial cells, thyroid cells, and many other cells; and 2) PD-L2, which is only expressed on the surface of macrophages and dendritic cells. The binding of PD-1 to its ligands will induce an inhibitory effect on cytotoxic T-cells activity by regulating their glucose metabolism (decreased glucose uptake and gluconeogenesis) and triggering their apoptosis, while also forestalling the co-stimulatory pathway of CD28-CD80/8^67-70^. Treg cells are the only cells escaping apoptosis as they are able to suppress cytotoxic CD8+T-cell proliferation, therefore supporting the immune escape of tumor cells^67,68,70,71^. It has been shown that PD-L1 are mostly present in inflammatory settings due to the fact that they are strongly regulated by interferon gamma^70,72^. Hence, chronic inflammation surrounding tumors could explain the limited destruction of cancer cells in such scenarios^70,73^. Furthermore, tumor aggressiveness has been shown to be directly proportional to the expression of PD-L1: the higher the PD-L1 expression, the greater the tumor aggressiveness^74^. CTLA-4, also known as CD152, is a co-stimulatory glycoprotein expressed on the surface of CD4+ and CD8+ T-cells^64^, which downregulates effector T-cells activation^68^. Despite its similarities to CD28, CTLA-4 has a 20-fold higher binding affinity to B7 glycoproteins: 1) B7.1 or CD80, and 2) B7.2 or CD86^68,69^. This limits activation of effector T-cells proliferation^5^, henceforth backing up the immune escape of tumor cells^70^. Both pathways are significant modulators of immune-tumor interaction and targeting them focused significant energy in the past several years, with notable successes^43^. However, because they regulate different phases of the immune response (CTLA-4 regulates the early stages of T-cell activation, whereas PD-1 is expressed after T-cell activation) and act at different sites (tumor microenvironment for PD-1/PD-L1 and draining lymph nodes for CTLA-4), it is fathomable that their effects and adverse events differ^70,75,76^. Noteworthy, it has been shown that anti-PD-1 have a more specific effect, with less severe adverse events^70,75,77^. Stimulation of T-cells in the periphery with immunomodulators have also beneficial effects against CNS tumors. A recent study has shown that pembrolizumab-induced PD-1 inhibition results in 20-30% responses in CNS, in patients with melanoma or non-small lung cancer CNS metastasis. Moreover, combined regimen of nivolumab and ipilimumab, which acts by both inhibiting PD-1 and CTLA-4 has notable 55% CNS response in melanoma brain metastasis patients^43^. Additionally, radiation therapy (e.g., SRS) is known to sensitize melanoma brain metastases to the action of checkpoint inhibitors, such as ipilimumab^78^.

### 6.3 Advances in Immunotherapy

The first immunotherapeutic to show effect against melanoma brain metastases was high dose interleukin 2 (hdIL-2). Melanoma patients with CNS involvement require higher doses of IL-2, which is challenging due to adverse events such as neurotoxicities and the need for hydration to counteract the induced vasodilation^30,79^. Recently, several immunomodulatory drugs were approved for melanoma treatment, with a recent study showing that the immune checkpoint blocking immunotherapy can double survival rates for patients with melanoma brain metastases^5^. Patients receiving these immunomodulatory drugs showed a mean survival of ~12.5 months compared to ~5.2 months for those not receiving immunotherapy, with a 4-year survival of ~28% versus only ~11%^5,80^.

### 6.4 Clinical Trials

It is important to point out that, currently, there are several clinical trials underway for melanoma brain metastases. Clinical trials are research studies conducted in volunteers and designed to evaluate the efficacy of new interventions. According to the general rules, any clinical study, including clinical trials in patients with brain metastases, needs to follow a strict protocol established prior to the beginning of the study. These protocols will specify the eligibility criteria, the number of participants, the length of the study, whether there will be a control group or any other way to limit research bias, the posology and route of administration, and the method of data analysis. Due to high mortality rates in patients with melanoma brain metastases, there is a pressing need for the discovery of new agents to effectively treat patients who have failed standard therapies. In the past, patients with brain metastases have been excluded from clinical trials, however their inclusion has been rising nowadays^81^. And the discovery of immunomodulatory drugs led to the development of many clinical studies targeting such patients. The results of already finished clinical trials have shown that immunotherapy significantly prolongs survival in patients with previously untreated melanoma brain metastases. The combination of CTLA-4 inhibitor ipilimumab with the PD-1 inhibitor nivolumab is the preferred treatment modality for patients with asymptomatic, untreated brain metastases from melanoma. Data supporting its use in this population comes from an open-label single-arm phase II trial (CheckMate-204), in which 101 patients were treated. This combination demonstrated an intracranial clinical benefit of 57%, which was superior to previously reported with ipilimumab (24%) or nivolumab (22%) alone, and with ipilimumab plus fotemustine (50%). The rate of adverse events associated with these agents was similar between the group tested and patients without brain metastases, with a low percentage of severe neurotoxicity^44,82^. The ABC phase II trial compared combination immunotherapy with single-agent nivolumab in 60 asymptomatic patients with no prior treatment, again showing a higher rate of intracranial response with the combination (46%) than with nivolumab alone (20%)^44,83^. To further consolidate such findings, another randomized phase III trial (NIBIT-M2) including 80 patients with untreated asymptomatic brain metastases, demonstrated a higher overall-survival rate in patients treated with combination nivolumab plus ipilimumab (29.2 months), than those treated with fotemustine alone (8.5 months) or in combination with ipilimumab (8.2 months)^44,84^. For symptomatic brain metastases from melanoma, the available data regarding the efficacy of immunotherapy as a single prime therapy is very limited. Such patients often require glucocorticoids, surgical resection and/or SRS to treat neurological symptoms prior to beginning immunotherapy^44^.

### 6.5 Limitations

Immunomodulatory drugs, such as PD-1/PD-L1 or CTLA-4 inhibitors, have a great therapeutic potential in metastatic melanoma, including melanoma brain metastases. Yet, only a small percentage of the patients are actually responding to these immunotherapies, with a high percentage of resistant cases. An extensive understanding of these mechanisms and causes of resistance for brain metastases is required in order to overcome this resistance.

One limitation to these investigations is the current methods used to investigate the tumor and in situ tumor microenvironment of the brain, which provide limited information of a heterogeneous tissue, spatially and dynamically, in time^51^. Another limitation is the lack of preclinical models which can mimic with high accuracy human brain metastases and that can recapitulate all the steps of brain metastases development^46^. As some research groups

suggest, the development of intravital microscopy technologies for high resolution imaging of brain metastases can be an important step forward^51^. The lack of predictive biomarkers of response and toxicity is another limitation of immunotherapy^10^. Additionally, some treated patients with brain metastases may need control of their symptoms with steroids, which can make immunotherapy ineffective^43^.

Taking into consideration these facts, significant research has to be further performed in order to clearly define which patients respond to immune checkpoint inhibitors and how to sensitize the non-responders to these therapies.

### 6.6 Pseudoprogression

A small number of patients with melanoma brain metastases experience pseudoprogression at the time of immunotherapy. Even though there is still no agreement on its precise molecular mechanism, it is believed to result from an invasion of lymphocytes leading to the formation of new tumor lesions, or the growth of existing ones, before their subsequent regression during continued therapy (or rarely, after discontinued treatment).

**Figure 3:**
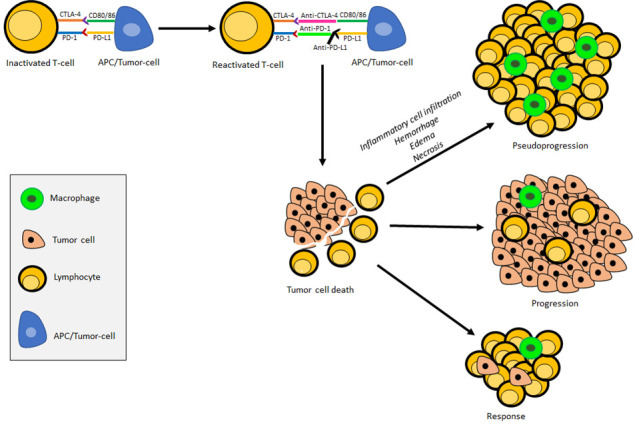
Pseudoprogression following Immunotherapy Immune checkpoint inhibitors reactivate T-cells heretofore inactivated by PD-L1 and CTLA-4 presentation by tumor cells or antigen-presenting cells (APCs), thus allowing them to successively invade and kill tumor lesions. The concomitant occurrence of inflammatory cell infiltration, hemorrhage, edema and necrosis promotes gradual lesion expansion as seen in imaging methods, thus indicating pseudoprogression.

At first, T-cells are inactivated subsequent to PD-L1 and CTLA-4 presentation by tumor cells or antigen-presenting cells (APCs). They are then reactivated following the administration of immune checkpoint inhibitors, namely anti-PD-1/PD-L1/CTLA-4. Activated T-cells will successively invade and kill tumor lesions, resulting in the release of antigens as they die, which attracts more inflammatory cells. Tumor shrinkage can lead to rupture of blood vessels and the formation of hemorrhages in locoregional lesions, which can lead to edema of the lesions along with an inflammatory response. Moreover, as the necrotic byproducts of dead tumor cells cannot be immediately absorbed, they accumulate in locoregional lesions. Therefore, the concomitant occurrence of inflammatory cell infiltration, hemorrhage, edema and necrosis causes gradual lesion expansion as seen in imageology studies, thus indicating pseudoprogression (Figure 3)^85^.

Pseudoprogression can be difficult to distinguish from true progression given the similarity of symptoms. As a result, the clinical treatment becomes more difficult, and patients and their families may get confused. Because immunotherapy is a relatively new treatment, there is limited data to guide clinical decision-making in such patients^30,86^.

## 7. Conclusion

Brain metastases are about 10 times more frequent than a primary brain tumor, being present in 20-40% of adults with systemic cancer. Malignant melanoma is the deadliest form of skin cancer, and its worldwide incidence has been increasing over the years. Advanced melanoma is well known for its propensity to metastasize to the brain and patients diagnosed with melanoma brain metastases have an overall survival of only 4 to 6 months with standard available treatments, such as surgery and radiation therapy. This is definitely not the desired outcome and sustained efforts are currently underway to develop better therapies.

Immunotherapy brings great promise as new tools for melanoma treatment, in particular, and for the treatment of other types of malignancies in general. This new modality is able to at least double the overall survival rates for patients with melanoma brain metastases. However promising, they require additional investigation. It is now imperative to detect better biomarkers within the CNS which can guide the therapeutic strategy and can predict the response to therapy. Although there has been great progress in recent years, there are still many challenges and limitations to overcome, and thus, a need to investigate, understand, and develop effective therapies to treat patients with melanoma brain metastases in a cost-effective manner with greater value to patients.

## References

[1] Langley Robert R, Fidler Isaiah J (2013). The biology of brain metastasis.. Clinical chemistry.

[2] Lassman Andrew B, DeAngelis Lisa M (2003). Brain metastases.. Neurologic clinics.

[3] Davies Michael A, Liu Ping, McIntyre Susan, Kim Kevin B, Papadopoulos Nicholas, Hwu Wen-Jen, Hwu Patrick, Bedikian Agop (2011). Prognostic factors for survival in melanoma patients with brain metastases.. Cancer.

[4] Jindal Vishal, Gupta Sorab (2018). Expected Paradigm Shift in Brain Metastases Therapy—Immune Checkpoint Inhibitors. Molecular Neurobiology.

[5] Iorgulescu J. Bryan, Harary Maya, Zogg Cheryl K., Ligon Keith L., Reardon David A., Hodi F. Stephen, Aizer Ayal A., Smith Timothy R. (2018). Improved Risk-Adjusted Survival for Melanoma Brain Metastases in the Era of Checkpoint Blockade Immunotherapies: Results from a National Cohort. Cancer Immunology Research.

[6] (2019). How Immunotherapy Is Used to Treat Cancer. American Cancer Society; Accessed June 13, 2020..

[7] Soare Georgiana R., Soare Costin A. (2019). Immunotherapy for Breast Cancer: First FDA Approved Regimen. Discoveries.

[8] Chang Liisa, Chang Minna, Chang Hanna M., Chang Fuju (2018). Microsatellite Instability: A Predictive Biomarker for Cancer Immunotherapy. Applied Immunohistochemistry &amp; Molecular Morphology.

[9] Bertrand J, Steingrimsson E, Jouenne F, Paillerets B, Larue L (2020). Melanoma Risk and Melanocyte Biology. Acta Dermato Venereologica.

[10] De Vellis Chiara, Pietrobono Silvia, Stecca Barbara (2021). The Role of Glycosylation in Melanoma Progression. Cells.

[11] Paluncic Jasmina, Kovacevic Zaklina, Jansson Patric J., Kalinowski Danuta, Merlot Angelika M., Huang Michael L.-H., Lok Hiu Chuen, Sahni Sumit, Lane Darius J.R., Richardson Des R. (2016). Roads to melanoma: Key pathways and emerging players in melanoma progression and oncogenic signaling. Biochimica et Biophysica Acta (BBA) - Molecular Cell Research.

[12] (2020). Cancer Statistics. NATIONAL CANCER INSTITUTE; Accessed January 13, 2023..

[13] Miller Kimberly D., Fidler‐Benaoudia Miranda, Keegan Theresa H., Hipp Heather S., Jemal Ahmedin, Siegel Rebecca L. (2020). Cancer statistics for adolescents and young adults, 2020. CA: A Cancer Journal for Clinicians.

[14] Zaidi MR, Fisher DE, Rizos Helen (2020). Biology of Melanocytes and Primary Melanoma..

[15] Westphal Dana, Glitza Oliva Isabella C, Niessner Heike (2017). Molecular insights into melanoma brain metastases.. Cancer.

[16] Abate-Daga Daniel, Ramello Maria C, Smalley Inna, Forsyth Peter A, Smalley Keiran S M (2018). The biology and therapeutic management of melanoma brain metastases.. Biochemical pharmacology.

[17] Köhler Corinna, Nittner David, Rambow Florian, Radaelli Enrico, Stanchi Fabio, Vandamme Niels, Baggiolini Arianna, Sommer Lukas, Berx Geert, van den Oord Joost J, Gerhardt Holger, Blanpain Cedric, Marine Jean-Christophe (2017). Mouse Cutaneous Melanoma Induced by Mutant BRaf Arises from Expansion and Dedifferentiation of Mature Pigmented Melanocytes.. Cell stem cell.

[18] Leupold Dieter, Pfeifer Lutz, Hofmann Maja, Forschner Andrea, Wessler Gerd, Haenssle Holger (2021). From Melanocytes to Melanoma Cells: Characterization of the Malignant Transformation by Four Distinctly Different Melanin Fluorescence Spectra (Review).. International journal of molecular sciences.

[19] Lucianò Anna Maria, Pérez-Oliva Ana B, Mulero Victoriano, Del Bufalo Donatella (2021). Bcl-xL: A Focus on Melanoma Pathobiology.. International journal of molecular sciences.

[20] Di Micco Raffaella, Krizhanovsky Valery, Baker Darren, d'Adda di Fagagna Fabrizio (2021). Cellular senescence in ageing: from mechanisms to therapeutic opportunities.. Nature reviews. Molecular cell biology.

[21] Davis Elizabeth J, Johnson Douglas B, Sosman Jeffrey A, Chandra Sunandana (2018). Melanoma: What do all the mutations mean?. Cancer.

[22] Shtivelman Emma, Davies Michael Q A, Hwu Patrick, Yang James, Lotem Michal, Oren Moshe, Flaherty Keith T, Fisher David E (2014). Pathways and therapeutic targets in melanoma.. Oncotarget.

[23] Patchell Roy A (2003). The management of brain metastases.. Cancer treatment reviews.

[24] Johnson John D., Young Byron (1996). Demographics of Brain Metastasis. Neurosurgery Clinics of North America.

[25] Farber S. Harrison, Tsvankin Vadim, Narloch Jessica L., Kim Grace J., Salama April K. S., Vlahovic Gordana, Blackwell Kimberly L., Kirkpatrick John P., Fecci Peter E. (2016). Embracing rejection: Immunologic trends in brain metastasis. OncoImmunology.

[26] Cruz-Muñoz William, Kerbel Robert S. (2011). Preclinical approaches to study the biology and treatment of brain metastases. Seminars in Cancer Biology.

[27] Kircher David, Silvis Mark, Cho Joseph, Holmen Sheri (2016). Melanoma Brain Metastasis: Mechanisms, Models, and Medicine. International Journal of Molecular Sciences.

[28] Fein Miriam R., Egeblad Mikala (2013). Caught in the act: revealing the metastatic process by live imaging. Disease Models &amp; Mechanisms.

[29] Quail Daniela F, Joyce Johanna A (2013). Microenvironmental regulation of tumor progression and metastasis. Nature Medicine.

[30] Margolin K K, Davies M, Kluger H, Tawbi H (2020). Melanoma Brain Metastasis: Unique Biology and Implications for Systemic Therapy.

[31] Redmer Torben (2018). Deciphering mechanisms of brain metastasis in melanoma - the gist of the matter.. Molecular cancer.

[32] Wilhelm Imola, Molnár Judit, Fazakas Csilla, Haskó János, Krizbai István A (2013). Role of the blood-brain barrier in the formation of brain metastases.. International journal of molecular sciences.

[33] Chen Qing, Boire Adrienne, Jin Xin, Valiente Manuel, Er Ekrem Emrah, Lopez-Soto Alejandro, Jacob Leni, Patwa Ruzeen, Shah Hardik, Xu Ke, Cross Justin R, Massagué Joan (2016). Carcinoma-astrocyte gap junctions promote brain metastasis by cGAMP transfer.. Nature.

[34] Hajal Cynthia, Shin Yoojin, Li Leanne, Serrano Jean Carlos, Jacks Tyler, Kamm Roger D (2021). The CCL2-CCR2 astrocyte-cancer cell axis in tumor extravasation at the brain.. Science advances.

[35] Proietti Ilaria, Skroza Nevena, Michelini Simone, Mambrin Alessandra, Balduzzi Veronica, Bernardini Nicoletta, Marchesiello Anna, Tolino Ersilia, Volpe Salvatore, Maddalena Patrizia, Di Fraia Marco, Mangino Giorgio, Romeo Giovanna, Potenza Concetta (2020). BRAF Inhibitors: Molecular Targeting and Immunomodulatory Actions.. Cancers.

[36] Aspelund Aleksanteri, Antila Salli, Proulx Steven T, Karlsen Tine Veronica, Karaman Sinem, Detmar Michael, Wiig Helge, Alitalo Kari (2015). A dural lymphatic vascular system that drains brain interstitial fluid and macromolecules.. The Journal of experimental medicine.

[37] Louveau Antoine, Smirnov Igor, Keyes Timothy J, Eccles Jacob D, Rouhani Sherin J, Peske J David, Derecki Noel C, Castle David, Mandell James W, Lee Kevin S, Harris Tajie H, Kipnis Jonathan (2015). Structural and functional features of central nervous system lymphatic vessels.. Nature.

[38] Louveau Antoine, Harris Tajie H, Kipnis Jonathan (2015). Revisiting the Mechanisms of CNS Immune Privilege.. Trends in immunology.

[39] Weiss Nicolas, Miller Florence, Cazaubon Sylvie, Couraud Pierre-Olivier (2009). The blood-brain barrier in brain homeostasis and neurological diseases.. Biochimica et biophysica acta.

[40] Berghoff Anna S, Fuchs Elisabeth, Ricken Gerda, Mlecnik Bernhard, Bindea Gabriela, Spanberger Thomas, Hackl Monika, Widhalm Georg, Dieckmann Karin, Prayer Daniela, Bilocq Amelie, Heinzl Harald, Zielinski Christoph, Bartsch Rupert, Birner Peter, Galon Jerome, Preusser Matthias (2016). Density of tumor-infiltrating lymphocytes correlates with extent of brain edema and overall survival time in patients with brain metastases.. Oncoimmunology.

[41] Quail Daniela F., Joyce Johanna A. (2017). The Microenvironmental Landscape of Brain Tumors. Cancer Cell.

[42] Osswald Matthias, Jung Erik, Sahm Felix, Solecki Gergely, Venkataramani Varun, Blaes Jonas, Weil Sophie, Horstmann Heinz, Wiestler Benedikt, Syed Mustafa, Huang Lulu, Ratliff Miriam, Karimian Jazi Kianush, Kurz Felix T, Schmenger Torsten, Lemke Dieter, Gömmel Miriam, Pauli Martin, Liao Yunxiang, Häring Peter, Pusch Stefan, Herl Verena, Steinhäuser Christian, Krunic Damir, Jarahian Mostafa, Miletic Hrvoje, Berghoff Anna S, Griesbeck Oliver, Kalamakis Georgios, Garaschuk Olga, Preusser Matthias, Weiss Samuel, Liu Haikun, Heiland Sabine, Platten Michael, Huber Peter E, Kuner Thomas, von Deimling Andreas, Wick Wolfgang, Winkler Frank (2015). Brain tumour cells interconnect to a functional and resistant network.. Nature.

[43] Kamath Suneel D, Kumthekar Priya U (2018). Immune Checkpoint Inhibitors for the Treatment of Central Nervous System (CNS) Metastatic Disease.. Frontiers in oncology.

[44] Samlowski W.E. (2023). Management of Brain Metastases in Melanoma.

[45] Tarhini AA, Agarwala SS, Khunger A, Wahl RL, Balch CM (2020). Diagnosis of Stage IV Melanoma..

[46] Puhalla S., Elmquist W., Freyer D., Kleinberg L., Adkins C., Lockman P., McGregor J., Muldoon L., Nesbit G., Peereboom D., Smith Q., Walker S., Neuwelt E. (2015). Unsanctifying the sanctuary: challenges and opportunities with brain metastases. Neuro-Oncology.

[47] Jenkins Russell W., Fisher David E. (2021). Treatment of Advanced Melanoma in 2020 and Beyond. Journal of Investigative Dermatology.

[48] West Howard (Jack), Jin Jill O. (2015). Performance Status in Patients With Cancer. JAMA Oncology.

[49] Lin Xuling, DeAngelis Lisa M. (2015). Treatment of Brain Metastases. Journal of Clinical Oncology.

[50] Staudt M, Lasithiotakis K, Leiter U, Meier F, Eigentler T, Bamberg M, Tatagiba M, Brossart P, Garbe C (2010). Determinants of survival in patients with brain metastases from cutaneous melanoma. British Journal of Cancer.

[51] Owyong Mark, Hosseini-Nassab Niloufar, Efe Gizem, Honkala Alexander, van den Bijgaart Renske J.E., Plaks Vicki, Smith Bryan Ronain (2017). Cancer Immunotherapy Getting Brainy: Visualizing the Distinctive CNS Metastatic Niche to Illuminate Therapeutic Resistance. Drug Resistance Updates.

[52] Cohen Justine V., Kluger Harriet M. (2016). Systemic Immunotherapy for the Treatment of Brain Metastases. Frontiers in Oncology.

[53] Nowak-Sadzikowska Jadwiga, Walasek Tomasz, Jakubowicz Jerzy, Blecharz Paweł, Reinfuss Marian (2016). Current treatment options of brain metastases and outcomes in patients with malignant melanoma. Reports of Practical Oncology &amp; Radiotherapy.

[54] Yamamoto Masaaki, Serizawa Toru, Shuto Takashi, Akabane Atsuya, Higuchi Yoshinori, Kawagishi Jun, Yamanaka Kazuhiro, Sato Yasunori, Jokura Hidefumi, Yomo Shoji, Nagano Osamu, Kenai Hiroyuki, Moriki Akihito, Suzuki Satoshi, Kida Yoshihisa, Iwai Yoshiyasu, Hayashi Motohiro, Onishi Hiroaki, Gondo Masazumi, Sato Mitsuya, Akimitsu Tomohide, Kubo Kenji, Kikuchi Yasuhiro, Shibasaki Toru, Goto Tomoaki, Takanashi Masami, Mori Yoshimasa, Takakura Kintomo, Saeki Naokatsu, Kunieda Etsuo, Aoyama Hidefumi, Momoshima Suketaka, Tsuchiya Kazuhiro (2014). Stereotactic radiosurgery for patients with multiple brain metastases (JLGK0901): a multi-institutional prospective observational study. The Lancet Oncology.

[55] Glitza Oliva Isabella, Tawbi Hussein, Davies Michael A. (2017). Melanoma Brain Metastases. The Cancer Journal.

[56] Chapman Paul B., Hauschild Axel, Robert Caroline, Haanen John B., Ascierto Paolo, Larkin James, Dummer Reinhard, Garbe Claus, Testori Alessandro, Maio Michele, Hogg David, Lorigan Paul, Lebbe Celeste, Jouary Thomas, Schadendorf Dirk, Ribas Antoni, O'Day Steven J., Sosman Jeffrey A., Kirkwood John M., Eggermont Alexander M.M., Dreno Brigitte, Nolop Keith, Li Jiang, Nelson Betty, Hou Jeannie, Lee Richard J., Flaherty Keith T., McArthur Grant A. (2011). Improved Survival with Vemurafenib in Melanoma with BRAF V600E Mutation. New England Journal of Medicine.

[57] Hauschild Axel, Grob Jean-Jacques, Demidov Lev V, Jouary Thomas, Gutzmer Ralf, Millward Michael, Rutkowski Piotr, Blank Christian U, Miller Wilson H, Kaempgen Eckhart, Martín-Algarra Salvador, Karaszewska Boguslawa, Mauch Cornelia, Chiarion-Sileni Vanna, Martin Anne-Marie, Swann Suzanne, Haney Patricia, Mirakhur Beloo, Guckert Mary E, Goodman Vicki, Chapman Paul B (2012). Dabrafenib in BRAF-mutated metastatic melanoma: a multicentre, open-label, phase 3 randomised controlled trial. The Lancet.

[58] Chen Guo, Davies Michael A. (2012). Emerging insights into the molecular biology of brain metastases. Biochemical Pharmacology.

[59] McQuade Jennifer, Davies Michael A (2015). Converting biology into clinical benefit: lessons learned from BRAF inhibitors. Melanoma Management.

[60] Postow Michael A., Callahan Margaret K., Barker Christopher A., Yamada Yoshiya, Yuan Jianda, Kitano Shigehisa, Mu Zhenyu, Rasalan Teresa, Adamow Matthew, Ritter Erika, Sedrak Christine, Jungbluth Achim A., Chua Ramon, Yang Arvin S., Roman Ruth-Ann, Rosner Samuel, Benson Brenna, Allison James P., Lesokhin Alexander M., Gnjatic Sacha, Wolchok Jedd D. (2012). Immunologic Correlates of the Abscopal Effect in a Patient with Melanoma. New England Journal of Medicine.

[61] Fernandes Gil Nuno Castro (2019). Immunotherapy for Melanoma Brain Metastases. Discoveries.

[62] Hou Wanzhu, Xu Guangpi, Wang Hanjie (2011). Basic immunology and immune system disorders. Treating Autoimmune Disease with Chinese Medicine.

[63] Spain Lavinia, Diem Stefan, Larkin James (2016). Management of toxicities of immune checkpoint inhibitors. Cancer Treatment Reviews.

[64] Wright Jordan J., Powers Alvin C., Johnson Douglas B. (2021). Endocrine toxicities of immune checkpoint inhibitors. Nature Reviews Endocrinology.

[65] Ferrari Silvia Martina, Fallahi Poupak, Galetta Fabio, Citi Emanuele, Benvenga Salvatore, Antonelli Alessandro (2018). Thyroid disorders induced by checkpoint inhibitors. Reviews in Endocrine and Metabolic Disorders.

[66] Shevach Ethan M. (2011). Biological Functions of Regulatory T Cells. Advances in Immunology.

[67] Francisco Loise M., Sage Peter T., Sharpe Arlene H. (2010). The PD-1 pathway in tolerance and autoimmunity. Immunological Reviews.

[68] Ferrari Silvia Martina, Fallahi Poupak, Elia Giusy, Ragusa Francesca, Ruffilli Ilaria, Patrizio Armando, Galdiero Maria Rosaria, Baldini Enke, Ulisse Salvatore, Marone Gianni, Antonelli Alessandro (2019). Autoimmune Endocrine Dysfunctions Associated with Cancer Immunotherapies. International Journal of Molecular Sciences.

[69] Paschou S.A., Stefanaki K., Psaltopoulou T., Liontos M., Koutsoukos K., Zagouri F., Lambrinoudaki I., Dimopoulos M.-A. (2021). How we treat endocrine complications of immune checkpoint inhibitors. ESMO Open.

[70] Chera Alexandra, Stancu Andreea Lucia, Bucur Octavian (2022). Thyroid-related adverse events induced by immune checkpoint inhibitors. Frontiers in Endocrinology.

[71] Chye Adrian, Allen India, Barnet Megan, Burnett Deborah L. (2022). Insights Into the Host Contribution of Endocrine Associated Immune-Related Adverse Events to Immune Checkpoint Inhibition Therapy. Frontiers in Oncology.

[72] Garcia-Diaz Angel, Shin Daniel Sanghoon, Moreno Blanca Homet, Saco Justin, Escuin-Ordinas Helena, Rodriguez Gabriel Abril, Zaretsky Jesse M., Sun Lu, Hugo Willy, Wang Xiaoyan, Parisi Giulia, Saus Cristina Puig, Torrejon Davis Y., Graeber Thomas G., Comin-Anduix Begonya, Hu-Lieskovan Siwen, Damoiseaux Robert, Lo Roger S., Ribas Antoni (2017). Interferon Receptor Signaling Pathways Regulating PD-L1 and PD-L2 Expression. Cell Reports.

[73] Imblum Brittney A., Baloch Zubair W., Fraker Douglas, LiVolsi Virginia A. (2019). Pembrolizumab-Induced Thyroiditis. Endocrine Pathology.

[74] Chowdhury Subrata, Veyhl Joe, Jessa Fatima, Polyakova Olena, Alenzi Ahmed, MacMillan Christina, Ralhan Ranju, Walfish Paul G. (2016). Programmed death-ligand 1 overexpression is a prognostic marker for aggressive papillary thyroid cancer and its variants. Oncotarget.

[75] Pardoll Drew M. (2012). The blockade of immune checkpoints in cancer immunotherapy. Nature Reviews Cancer.

[76] Varricchi Gilda, Loffredo Stefania, Marone Giancarlo, Modestino Luca, Fallahi Poupak, Ferrari Silvia Martina, de Paulis Amato, Antonelli Alessandro, Galdiero Maria Rosaria (2019). The Immune Landscape of Thyroid Cancer in the Context of Immune Checkpoint Inhibition. International Journal of Molecular Sciences.

[77] Okazaki T., Honjo T. (2007). PD-1 and PD-1 ligands: from discovery to clinical application. International Immunology.

[78] Knisely Jonathan P. S., Yu James B., Flanigan Jaclyn, Sznol Mario, Kluger Harriet M., Chiang Veronica L. S. (2012). Radiosurgery for melanoma brain metastases in the ipilimumab era and the possibility of longer survival. Journal of Neurosurgery.

[79] Atkins Michael B., Lotze Michael T., Dutcher Janice P., Fisher Richard I., Weiss Geoffrey, Margolin Kim, Abrams Jeff, Sznol Mario, Parkinson David, Hawkins Michael, Paradise Carolyn, Kunkel Lori, Rosenberg Steven A. (1999). High-Dose Recombinant Interleukin 2 Therapy for Patients With Metastatic Melanoma: Analysis of 270 Patients Treated Between 1985 and 1993. Journal of Clinical Oncology.

[80] (2018). Immunotherapy doubles survival rates for patients with melanoma brain metastases.. Brigham and Women’s Hospital. Medical Xpress. Accessed January 14, 2022..

[81] Abrey LE (2011). Inclusion of patients with brain metastases in clinical trials.. Open Access Journals. 2011;1(8):1065-1068. Accessed January 14, 2023..

[82] Tawbi Hussein A., Forsyth Peter A., Algazi Alain, Hamid Omid, Hodi F. Stephen, Moschos Stergios J., Khushalani Nikhil I., Lewis Karl, Lao Christopher D., Postow Michael A., Atkins Michael B., Ernstoff Marc S., Reardon David A., Puzanov Igor, Kudchadkar Ragini R., Thomas Reena P., Tarhini Ahmad, Pavlick Anna C., Jiang Joel, Avila Alexandre, Demelo Sheena, Margolin Kim (2018). Combined Nivolumab and Ipilimumab in Melanoma Metastatic to the Brain. New England Journal of Medicine.

[83] Long G.V., Atkinson V.G., Lo S., Sandhu S.K., Brown M., Gonzalez M., Guminski A., Scolyer R.A., Emmett L., Menzies A.M., McArthur G.A. (2019). Long-term outcomes from the randomized phase II study of nivolumab (nivo) or nivo+ipilimumab (ipi) in patients (pts) with melanoma brain metastases (mets): Anti-PD1 brain collaboration (ABC). Annals of Oncology.

[84] Di Giacomo A.M., Sileni V. Chiarion, Del Vecchio M., Ferrucci P.F., Guida M., Quaglino P., Guidoboni M., Marchetti P., Cutaia O., Amato G., Gambale E., Calabrò L., Valente M., Danielli R., Giannarelli D., Mandala M., Maio M. (2020). 1081MO Efficacy of ipilimumab plus nivolumab or ipilimumab plus fotemustine vs fotemustine in patients with melanoma metastatic to the brain: Primary analysis of the phase III NIBIT-M2 trial. Annals of Oncology.

[85] Jia Wenxiao, Gao Qianqian, Han Anqin, Zhu Hui, Yu Jinming (2019). The potential mechanism, recognition and clinical significance of tumor pseudoprogression after immunotherapy. Cancer Biology &amp; Medicine.

[86] Simard Jillian L., Smith Melanie, Chandra Sunandana (2018). Pseudoprogression of Melanoma Brain Metastases. Current Oncology Reports.

